# Cognitive impairment assessment in schizophrenia. purposely a case

**DOI:** 10.1192/j.eurpsy.2024.1572

**Published:** 2024-08-27

**Authors:** N. Ogando Portilla, S. M. Bañón González, B. Gamo Bravo, M. E. González Laynez, M. A. Urbanos, M. M. Cortés

**Affiliations:** ^1^Psychiatry, Hospital Universitario Infanta Sofía, Madrid; ^2^Psychiatry, Hospital universitario de Toledo, Toledo; ^3^Psychiatry, Hospital Universitario La Princesa, Madrid; ^4^Psychiatry, Hospital General Universitario de Alicante Dr Balmis, Alicante, Spain

## Abstract

**Introduction:**

Significant and measurable cognitive symptoms are present at the onset of the disorder and these remain stable in the subsequent period between 2 and 5 years. Their deterioration increases with the course of the disease. Attention, concentration, psychomotor speed and resolution of conceptual tasks are usually affected and are more significant in the presence of positive symptoms.

**Objectives:**

Sometimes, the typical positive or negative symptoms of the disease do not adequately reflect the severity of cognitive impairment. Measuring this deterioration can be very relevant when evaluating the severity and the prognosis of the disorder.

**Methods:**

31-year-old male with a previous diagnosis of schizophrenia of 4 years of evolution. He gets a maintained treatment with amisulpiride 400mg with an apparent good response. A single hospitalization at the onset of the disease. An assassination attempt on his mother is done by suffocation with a pillow and observing a significant cognitive impairment despite an apparent control of the symptoms of schizophrenia.

**Results:**

An exhaustive neuropsychological evaluation is carried out, observing a very important cognitive deterioration that had not been previously detected and allowing a pharmacological adjustment of the underlying disease with global improvement of the patient.

**Conclusions:**

It is very important to evaluate the patient as a whole without forgetting the frequent cognitive damage that these patients can have. An intense neuropsychological study can be very useful to evaluate the prognosis and adequate treatment of the patient in order to reduce serious risks.

**Disclosure of Interest:**

None Declared

